# A mediation model of the relationship between university students’ news media literacy and xenophobia: The role of intellectual humility, perceived threat, and blind patriotism

**DOI:** 10.3389/fpsyg.2022.1036497

**Published:** 2022-11-08

**Authors:** Muyingnan Lin, Ching Sing Chai, Jyh-Chong Liang

**Affiliations:** ^1^Department of Curriculum and Instruction, Faculty of Education, The Chinese University of Hong Kong, Shatin, Hong Kong SAR, China; ^2^Program of Learning Sciences and Institute for Research Excellence in Learning Sciences, National Taiwan Normal University, Taipei, Taiwan

**Keywords:** news media literacy, xenophobia, intellectual humility, perceived threat, blind patriotism

## Abstract

The dissemination of misinformation and disinformation has increased the need for news media literacy. This study administered a self-developed questionnaire to measure the relationship between news media literacy and xenophobia among college students in China (*N* = 430). The questionnaire measured five variables: news media literacy, xenophobia, blind patriotism, perceived threat, and intellectual humility. Confirmatory factor analysis (CFA) was used to validate this five-variable survey, and the analyses indicated satisfactory construct validity. Results from structural equation modeling indicated that intellectual humility was a significant predictor of news media literacy, and blind patriotism and perceived threat mediated the relationship between news media literacy and xenophobia. This study provides insights for researchers and instructors who are promoting news media literacy education in schools.

## Introduction

News media literacy is an important form of literacy in the post-truth era in which social media are rife with various kinds of misinformation, and some news agencies work with politicians to advance their respective agendas. The post-truth era has not only threatened individuals’ ability to explore what is true (Barzilai and Chinn, 2020), but the accompanying mass dissemination of misinformation has also led to misperceptions and has influenced decision-making based on false beliefs, especially in terms of popularizing and perpetuating biases and ethnic stereotypes (Erba et al., 2019), as well as posing threats to individuals, organizations, and society as a whole (Talwar et al., 2019).

At present, although there is still a lack of clarity regarding the differences between the concepts of misinformation and disinformation, some preliminary consensus has been reached (Søe, 2017; Tucker et al., 2018). According to Born and Edgington (2017), misinformation is inaccurate information that is promulgated unintentionally, but it is intentionally neutral. In contrast, disinformation is spread knowingly to support the interests of individuals or organizations, and is motivated by a desire to destroy public confidence or to deceive or mislead. Therefore, it is reasonable to define the two notions as fully distinct concepts, which cannot simply be referred to as “fake news.”

Given the complexity of misleading information, people need to be careful with the information they receive. Affected by misleading information, people could be emotionally clouded and hence engage in behaviors that endanger themselves and others. Xenophobia might be one of the consequences. Previous studies on xenophobia have suggested its tremendous psychological impact on individuals and communities (Suleman et al., 2018). People with xenophobic beliefs have reported significant emotional distress, including vulnerability, depression, and loneliness (Vromans et al., 2011). In countries where xenophobia holds sway, incidents of violence happen repeatedly, and it seems to affect many people (Onah, 2008). On a global scale, the failure to address the issue of xenophobia threatens political transformation and further exacerbates irreconcilable national differences (De Master and Le Roy, 2000). Xenophobia warrants further attention as it threatens worldwide peace and harmony. By concluding the tragic events fueled by xenophobia during the COVID-19 pandemic, Mamun and Griffiths (2020) suggested that avoiding unreliable or untrustworthy sources of information might be beneficial for the public to reduce fear or panic about certain issues. Specifically, how people process and perceive information might influence their attitudes. Thus, news media literacy needs to be considered when discussing xenophobia.

The relationship between obtaining and perceiving information and some irrational behaviors or cognition is complex and could be influenced by multiple factors. The factors identified so far include hate speech exposure (Abdul et al., 2019), inherent stereotyping (Arendt, 2013), and political ideology (Tully et al., 2020). As Faulkner et al. (2004) suggested, there are many other personality factors or societal variables that may contribute to xenophobic attitudes. For example, the realistic group conflict theory and symbolic racism theory suggest that the perceived threat from an outgroup largely contributes to negative attitudes or feelings of prejudice toward those outgroups (Nshom et al., 2022). Symbolic politics theory proposes that the words and symbols political elites employ would increase negative stereotypes of specific groups and evoke emotional reactions (Fussell, 2014). In other words, emotional reactions depend on knowledge of a specific group and, therefore, can be manipulated to some extent by information people receive. Studies have also found that blind patriotism promotes preferences for a more homogeneous culture, and people who are blindly patriotic show more xenophobic reactions to some specific groups (Parker, 2010). These intergroup relations provide a more holistic view when exploring the relationship between news media literacy and xenophobia, including how one’s beliefs contribute to one’s information access, and what attitudes or behaviors one might have. Therefore, the roles of perceived threat and blind patriotism were also examined in this study.

Considering these issues, the importance of news media literacy is clear. However, published studies have analyzed only a limited number of factors that contribute to people’s news media literacy. Only need for cognition and media locus of control were found to be significant predictors (Su et al., 2022). Considering the importance of news media literacy, more potential factors need to be explored.

As a virtue, intellectual humility may promote news media literacy. According to Whitcomb et al. (2017), intellectual humility increases one’s propensity to admit one’s intellectual limitations to oneself and to others. It also helps one to consider alternative ideas, listen to the views of others, and spend more time trying to understand someone with whom one disagrees. These traits of intellectual humility encourage people to rationally access news content instead of being caught up in an echo chamber. A previous study suggested that people’s ratings of intellectual humility were associated with their information processing (Deffler et al., 2016). Therefore, the relationship between intellectual humility and news media literacy was explored in this study.

To clarify the situation and the underlying sociopsychological mechanisms involved in the relationship, this study focuses on the above three factors, namely intellectual humility, blind patriotism, and perceived threat. We gained empirical data from young adults in China to investigate: (1) whether intellectual humility may act as a predictor of news media literacy, (2) the relationship between news media literacy and xenophobia, and (3) whether the relationship between news media literacy and xenophobia is mediated by blind patriotism and perceived threat.

## Literature review and hypotheses development

### News media literacy

News media literacy refers to the knowledge and abilities needed to identify and engage with news (Maksl et al., 2015). Having news media literacy is important as it is the main competency that people need to rely on to avoid negativity bias (van der Meer and Hameleers, 2022). Recent research has revealed several long-established benefits of news media literacy. Maksl et al. (2015) reported that teenagers with higher levels of news media literacy were more critical of news reports and were likely more knowledgeable about current affairs. This may allow them to gain a good understanding of the media landscape, and help them to interact with the media to achieve pro-social and personal goals.

The significance of studying news media literacy has also been highlighted. As Ashley et al. (2013) suggested, cultivating news media literacy helps people to have a more holistic understanding of how news is produced. This knowledge equips people to better access, evaluate, and analyze news content. However, although a number of media literacy interventions have been developed in the past 30 years, meta-analytic assessment of the effects and factors which increase news literacy application are still unavailable (Jeong et al., 2012). In addition, there are insufficient studies on the contexts in which news literacy is performed (Swart, 2021). Thus, more studies on news media literacy would be beneficial.

Given the importance of news media literacy, researchers have found some educational ways to enhance it, such as integrating news and current affairs into instruction (Moore, 2013). Hobbs (2004) also noticed that some schools emphasize the importance of media issues studies, and students are taught to critically evaluate news content. Opportunities for media production are also offered in some places. However, researchers believe that educators have not grasped the whole picture of news media literacy.

Based on the above results, researchers have highlighted the need to explore potential factors which may have associations with news media literacy in future research, especially forming a model which includes multiple influences that might stimulate people to critically consume news (Tamboer et al., 2022). This could promote more effective initiatives to stimulate the involvement in current affairs. Although young people are called digital citizens and tend to be adept at using media as a way of obtaining information, their ability to examine media content is still limited (Musgrove et al., 2018), and little empirical evidence exists demonstrating the positive role of the new media literacy in assisting young people in discerning facts from falsehoods (Luo et al., 2022).

Considering the above suggestions, the present study focused on surveying young adults in Mainland China to explore the relationship between news media literacy, intellectual humility, and xenophobia. In addition, although news media literacy and related education are welcomed by the Chinese government, no educational policy has been developed in this field, and not many schools in China actually put news media literacy education into practice (Lee and Tiande, 2016). Therefore, this study also aimed to assess Chinese students’ news media literacy.

### Xenophobia

Xenophobia has become an important public issue in the context of cultural diversity. According to The United and Nations Organization (2001), xenophobia describes “attitudes, prejudices and behavior that reject, exclude and often vilify persons, based on the perception that they are outsiders or foreigners to the community, society or national identity” (p. 2). On the generation of xenophobia, Rydgren (2004) argued that xenophobic beliefs may be formed by stereotyping, as what people apprehend when confronting a new thing is contingent upon what they already know. Xenophobic values are prevalent in all societies, and there is no sign that such value will disappear (Hjerm, 2001). New concerns about xenophobia emerged during the COVID-19 pandemic as the debate and stigma over the origin of COVID-19 fueled xenophobia and even led to a “pandemic of hate” (Ittefaq et al., 2022).

Although some scholars believe that xenophobia should be regarded as an appeal for national and ethnic unity, the negative effects of xenophobia can still lead to considerable disastrous consequences. Studies on HIV-infected people found that xenophobia was one of the barriers for them to access medical care and other health assurance (Alvarez-del Arco et al., 2012). In Europe, increasing immigration has created problems for many Europeans, pushing some toward more conservative or extreme positions. De Master and Le Roy (2000) believed that these situations threaten the balanced political environment of the European Union. Hence, xenophobia constantly threatens the harmony of society and personal wellbeing. Unfortunately, xenophobia can be easily spread through social media.

Social media have been widely named as a major factor in the increase of expression of hate (Bursztyn et al., 2019), and it is clear that the emergence of modern media has influenced where and how xenophobia occurs, and its effect. For example, news websites’ comments have already become an important space for spreading hate speech (Erjavec and Kovačič, 2012). Nevertheless, due to the lack of news media literacy, people rely on low-quality information, and they are easily influenced by such media content. Specifically, as many people cannot process and perceive information objectively and rationally, the echo chambers created by news media fuel ideological homophily and give audience good reason for relying on them without much reflection. Political consumers in particular are increasingly turning to social media for information, but these sources are not beholden to traditional journalistic standards. More importantly, these sources play a deleterious role in shaping hostile attitudes toward specific groups or organizations (Lajevardi et al., 2022).

Although most studies on racism, nationalism, and xenophobia emphasize how biased messages and media influence audience attitudes, especially in inspiring favoritism for their own countries and questioning information provided by out-group media sources (Golan et al., 2021), much remains unclear. Firstly, although news media literacy seems to help people objectively access news content, the relationship between news media literacy and xenophobia has not been explored. Secondly, xenophobia has become an international phenomenon, but the previous measurements were mainly conducted in countries outside of Asia. Therefore, they need to be cross-culturally validated, with empirical data from China providing an Asian perspective on xenophobia. In this study, a specific xenophobia scale developed by van der Veer et al. (2011) was used to examine the relationship between news media literacy and xenophobia in China. Based on the above research gaps, hypothesis one was formulated as follows:

H1. News media literacy would be negatively associated with xenophobia.

### Blind patriotism

According to Parker (2010), blind patriotism is defined as an “uncritical support for national policies and practices” (p. 97). In other words, blind patriotism creates exclusive group boundaries, negative attitudes toward external groups, and denial of the nation’s transgressions against others (Schatz, 2020).

People who are biased with blind patriotism show different attitudes toward citizens and foreigners because they tend to fuse their personal identity with their social identity as in-group members. For example, Spry and Hornsey (2007) surveyed 95 Australians and found that people with higher scores on blind patriotism had more negative attitudes toward multiculturalism, immigration, and providing cultural services to immigrants. On the contrary, they had more positive attitudes toward cultural assimilation because they were worried about their national culture being “contaminated” by foreigners.

However, blind patriotism does not emerge from a vacuum; rather, it is related to the information people possess. By comparing two groups of college students who professed blind or constructive patriotism, researchers found that they had different types of information exposure (Parker et al., 2009). This finding was possibly because the bias in information access affects individual cognitive patterns. Moreover, stronger interpersonal bonds with fellow nationals can also predict a higher level of blind patriotism (Finell and Stevenson, 2022). This is because tight intergroup relationships may create an echo chamber, thus blocking group members from accepting diverse information.

Although research has found that prevalent ideologies and norms influence individuals’ attitudes toward other cultures, the role of blind patriotism has rarely been analyzed. This is especially so in China, where patriotism is different from that in Western countries (Hamada et al., 2021). Based on the above reasons, the Chinese geo-cultural context represents a large research gap. In addition, as nationalist historical beliefs are part of the structure of Chinese national identity (Gries et al., 2011), blind patriotism may profoundly affect the Chinese perception of the country’s problems, and this kind of orientation may help people to form subjective perceptions of the outside world.

Given the fact that blind patriotism is associated with obtaining and processing information, and might trigger irrational beliefs such as bias and stereotypes against out-groups, hypothesis two was posited:

H2. Blind patriotism would mediate the association between news media literacy and xenophobia.

### Perceived threat

According to the integrated threat theory, a threat happens when different groups perceive each other as competing for the allocation of scarce resources that are necessary for their security (Nshom et al., 2022). Hence, as Semyonov et al. (2004) concluded, perceived threat can be seen as a competitive threat to one’s actual interests and prerogatives, which in turn might increase hostility, antagonism, and discrimination against other groups.

As unfounded perceived threats may lead to many negative effects, researchers have explored the underlying mechanism. A study in Lebanon showed that there was a significant decrease in the number of vaccinated people due to exposure to false or verified news in the context of the COVID-19 pandemic (Ghaddar et al., 2022). These unconfirmed messages may convey a sense of unease or a potential threat, leading to public distrust in vaccines. That is to say, the information people access is important, as their cognition or behaviors might be highly influenced by that information. Calvillo et al.’s (2020) research involving 346 US citizens found that the perception of threat was related to which channel people obtained information from. For example, the researchers found that participants who got more news from Fox News would perceive less threat as they believed that the media were exaggerating the threat of the pandemic. However, participants who got more news from CNN would perceive more threat as they believed that COVID-19 was more severe. These findings suggest that there are relationships between perceived threats and the information that people receive. More specifically, what views people form about outgroup members or issues is related to how they understand information and what information they choose to believe.

Thus, focusing only on perceived threat might obscure our understanding of what triggers xenophobia since perceived threat mainly comes from the information people choose to believe. Therefore, the role of perceived threat should be considered in the relationship between news media literacy and xenophobia. Additionally, although previous research has found that blindly patriotic individuals or those who believe in an inflated image of an ingroup would perceive more threat from outgroups, samples are mainly from western countries (Spry and Hornsey, 2007; de Zavala et al., 2009; Willis-Esqueda et al., 2017). In China, researchers believe that Chinese people’s nationalist historical beliefs powerfully predict their perceptions of threats from foreign countries, which in turn directly affect their preferences for specific countries (Gries et al., 2011). Moreover, the study of public attitudes toward foreigners or immigrants can also contribute to the study of Chinese public opinion, which appears to play an increasingly important role in Chinese policy decision-making processes (Han, 2017).

Therefore, it is necessary to verify this relationship among non-western populations. Moreover, the conclusion that news media literacy might reduce the perception of fear or threat remains controversial, so the relationship between the two factors needs further investigation (Bergan and Lee, 2018). Therefore, hypotheses three and four were posited:

H3. Perceived threat would mediate the association between news media literacy and xenophobia.

H4. Blind patriotism would be positively associated with perceived threat.

### Intellectual humility

Intellectual humility is a cognitive virtue that can be defined as an individual’s acceptance of their personal limitations in adopting an absolute epistemic position (Leary et al., 2017). More specifically, it shows the degree to which an individual recognizes that a particular personal belief may be wrong (Deffler et al., 2016).

Intellectual humility could promote rational thinking. For example, samples from two American communities showed that intellectual humility predicts less political bias, helping individuals to escape from the echo chamber (Bowes et al., 2022). In the context of COVID-19, people with high intellectual humility are more likely to review online information rationally, rather than easily believing in misinformation (Koetke et al., 2022; Newman et al., 2022). These studies report the importance of intellectual humility in rationality development, which is also one of the significant components of news media literacy.

In addition, intellectual humility is related to the way individuals process information and think about their beliefs. Specifically, people with a high level of intellectual humility can recognize the limitations of evidence for their beliefs and are aware that their ability to acquire and evaluate relevant information is limited (Deffler et al., 2016). More importantly, having intellectual humility does not mean “being self-effacing or always yielding to others”; rather, it is a balance between dogmatically rejecting information that questions one’s views and giving in too quickly (Krumrei-Mancuso and Rouse, 2016). This evidence suggests that intellectual humility may be an important aspect when considering the formation of news media literacy.

Although previous findings suggested that intellectual humility is related to how people critically assess knowledge claims, there is not yet much research on the relationship between intellectual humility and individuals’ behaviors or cognition. Koetke et al. (2022) suggested that intellectual humility may provide some of the motivation behind engaging in behaviors that fight against misinformation as those who realize their tendency to make mistakes or be wrong may verify the accuracy of information more frequently to ensure that their beliefs will be accurate and objective. Thus, we suspect that intellectual humility is important for news media literacy and might be a predictor of it. Hence, hypothesis five is posited:

H5. Intellectual humility would be positively associated with news media literacy.

**FIGURE 1:**
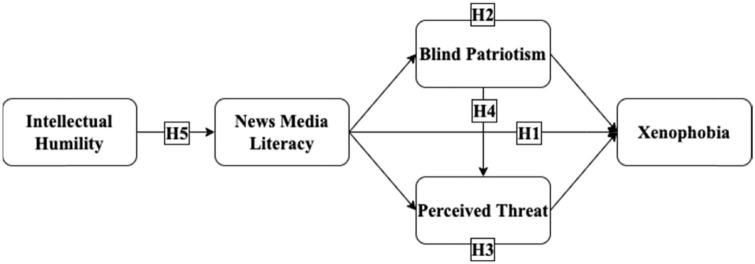
The hypothesized model.

Based on the above research assumptions, we formulated the following structural equation model (Figure 1). In this study, we hoped to clarify the relationship between news media literacy and xenophobia, and explore the role of intellectual humility, blind patriotism, and perceived threat in that relationship.

## Materials and methods

### Background

The present study’s geo-cultural context is China, where information is regulated by governmental control (Guo, 2020). Nonetheless, the prevalence of misinformation contributed by nearly two billion internet users inevitably makes China a post-truth society. Misinformation is accelerating and affecting the way individuals interpret daily affairs, which has resulted in serious societal and political consequences (Cheng and Lee, 2019; Guo, 2020). It is worth noting that news media literacy and xenophobia related studies have not been adequately conducted in China. Compared with developed Western countries, China’s news media literacy education is not universal, and knowledge of students’ levels of news media literacy is insufficient (Zhao, 2022). With the lack of news media knowledge, people may not realize that the press may serve as a “mouthpiece” (Zhang, 2021) for people in power. Therefore, it is necessary to understand the situations of news media literacy and xenophobia in China’s sociocultural and media environment.

In this study, we were curious about the relationship between news media literacy and xenophobia as information can affect public perceptions or shape human emotions (West, 2022). Also, we hypothesized that people with lower news media literacy may have higher blind patriotism and perceived threat, which might be the predictors of some extreme behaviors or cognition.

In this study, we recruited participants from Project 985 universities, Project 211 universities, and unranked universities. Most Project 985 universities are in the top 500 QS World University Rankings, while Project 211 universities are in the 500–1400th QS World University Rankings. The rest are listed as unranked universities as they have no world or national rankings. There is a significant gap in academic achievement between the universities of different levels. Therefore, students of these universities are also considered to have significant differences in their academic performances.

### Participants

A total of 430 participants (females = 197, males = 233) volunteered to take part in this research (Table 1); their average age was 22.28 years (*SD* = 0.13). The sample consisted of 104 top QS 500 university students, 107 middle-class university students, and 219 unranked university students. Of the 430, 191 were bachelor students, 217 were master’s students, and 22 were doctoral students. They were invited to complete an online survey at their own pace, and were compensated with a small monetary reward at the end of the study.

**TABLE 1:** The demographic characteristics of all the participants.

Variable		n (*N* = 430)	Proportion
Gender	Male	233	54.2%
	Female	197	45.8%
Age group (years)	18–20	101	23.5%
	21–23	248	57.7%
	24–26	60	14.1%
	27–29	14	3.3%
	30-Above	7	1.4%
Education	Bachelor’s degree	191	44.4%
	Master’s degree	217	50.5%
	Doctoral degree	22	5.1%
Academic performance	Top	104	24.2%
	Middle level	107	24.9%
	Low level	219	50.9%

### Instrument

#### Background factors

Age, gender, degree, and academic performance were selected as background factors. Although GPA has been widely used in defining students’ academic performance, the grading schemes of universities are different and hard to unify and standardize (Garipagaoglu et al., 2012). In order to avoid fallacy in the interpretation of the students’ academic performance, the university ranking was selected to identify their general academic standing in this study.

#### News media literacy scale

The news media literacy scale developed by Ashley et al. (2013) was selected to measure participants’ notion of the gap existing between representation and reality in media reporting. This is a nine-item scale including items such as “Individuals can find news sources that reflect their own political values,” “Two people might see the same news story and get different information from it,” and “News makes things more dramatic than they really are.” Participants were asked to use a Likert scale to indicate their level of agreement or disagreement with each statement (from 1 = “strongly disagree” to 7 = “strongly agree”). The overall Cronbach’s alpha values reported by Jones-Jang et al. (2021) and Stamps (2021) were 0.82 and 0.90, respectively, which indicated that the coefficient of internal consistency for the total scale was high. The reliability of the news media literacy scale in this study was 0.94.

#### Xenophobia

With reference to and modification of a prior research scale (van der Veer et al., 2011), xenophobia was assessed using five items. The respondents were asked to rate their agreement with five statements using a 7-point Likert scale (from 1 = “strongly disagree” to 7 = “strongly agree”) including “Immigrants cause increase in crimes,” “Interacting with foreigners makes me uneasy,” “I worry that foreigners may spread unusual diseases,” “I am afraid that in case of war or political tension, immigrants will be loyal to their country of origin,” and “I am afraid that our own culture will be lost with increase in immigration.” In this study, the xenophobia scale showed an acceptable reliability of 0.88.

#### Blind patriotism

Drawing from Schatz et al. (1999), four items were used to assess blind patriotism. Participants were asked to rate their agreement on a 7-point Likert scale ranging from 1 = “strongly disagree” to 7 = “strongly agree” to determine whether individuals love their country irrationally. Sample items included “I would support my country whether it was right or wrong.” The scale has been used with an Australian population (Spry and Hornsey, 2007) and an American population (Parker, 2010) and showed a high coefficient of internal consistency of 0.73 and 0.78, respectively. The scale showed a good reliability of 0.85 in this study.

#### Perceived threat

Each participant responded to the perceived threat scale to self-report their perception of threat from foreigners. This survey was developed and validated by Semyonov et al. (2004) and consists of four items that measure perceived threat (e.g., “Foreigners who live in China are a strain on the welfare system”). Each item uses a 7-point Likert scale from 1 = “strongly disagree” to 7 = “strongly agree” for participants to select from. The reliability of the perceived threat scale in this study was 0.83.

#### Intellectual humility

The general intellectual humility scale of Leary et al. (2017) was selected to measure the intrapersonal aspects of intellectual humility. Participants were asked to rate their agreement with six items from 1 = “strongly disagree” to 7 = “strongly agree.” The scale has been widely used and presented high Cronbach’s α = 0.89 and 0.84 among different groups (Bowes et al., 2022). The Cronbach’s α of this scale was 0.88 in this study.

As the original scales shown above were all written in English, we asked English major students to help translate them into simplified Chinese. The scales were reviewed by two professors who majored in Chinese and English language, respectively. We also provided items in both English and Chinese to ensure the accuracy and minimize the errors. Then five university students were recruited to take part in a pilot test. They were required to give comments on the statement of each item, including the differences in expressions between the Chinese and English versions and the clarity of the translations. After that, we revised the statements according to their feedback. Finally, five more university students were recruited to take part in the second round of the pilot test; they all gave positive feedback on the Chinese version.

### Data collection

An online survey was conducted to help us verify the double mediator hypothesized model we proposed. On the first page of the questionnaire, participants signed a consent form (IRB Reference No. EDU2022-055), and they were fully informed of the purpose, hypotheses, and conditions of this research. In addition, we emphasized that this survey was for academic research only, participation was completely voluntary, and participants might withdraw at any time. We collected data by using the Tencent Survey Platform,^1^ a well-known data service platform in China. Participants could open the survey link directly in WeChat, which created a convenient environment for our investigation.

### Data analysis

A series of statistical analyses was performed to examine the data. Firstly, Pearson correlation and Spearman’s rank correlation analysis were used to analyze the relationships between the factors. With reference to Huang et al. (2017), as the sample size of this study was large, the correlation analyses were employed with a 0.001 level of statistical significance to prevent any Type I errors. Secondly, indicator reliability, construct reliability, convergent validity, and discriminant validity were assessed by indices including factor loadings, Cronbach’s alpha coefficients, composite reliability (CR), average variation extracted (AVE), and heterotrait-monotrait ratio of correlations (HTMT). Confirmatory factor analysis (CFA) was performed using SPSS Amos 24.0 with 5,000 bias-corrected bootstrap samples and 95% confidence intervals (CIs). The SEM analysis was also used to examine all the relationships among the factors. In addition, indices including χ^2^/*df*, the adjusted goodness-of-fit statistic (AGFI), root mean square error of approximation (RMSEA), the non-normed fit index (NNFI), the comparative fit index (CFI), and the incremental fit index (IFI) were employed to assess the comprehensive goodness of fit of the research model. Next, User-defined estimands were used to test our hypothesized model.

Before the analyses, univariate and multivariate normality tests were conducted. Firstly, we noted that both skewness (range: from –0.46 to 0.10) and kurtosis (range: from –1.07 to –0.60) were satisfied with the required range. Secondly, to test the multivariate normality, we noted that the standardized multivariate kurtosis was –0.961, which means that the data conformed to multivariate normal distribution (Noar, 2003).

## Results

### Correlations between variables

Pearson correlation coefficients were calculated to investigate the correlations among continuous variables, such as news media literacy, xenophobia, blind patriotism, intellectual humility, and perceived threat. Spearman’s rank correlation coefficient was also used to investigate the correlations between ordinal variables (university and degree) and other variables. As noted in Table 2, news media literacy is positively associated with intellectual humility, but it is negatively associated with blind patriotism, perceived threat, and xenophobia. On the other hand, blind patriotism is positively associated with perceived threat, and both factors are positively associated with xenophobia.

**TABLE 2:** Correlation between variables included in the study (*N* = 430).

	1	2	3	4	5	6	7	8
Age	−							
Gender	–0.021	−						
University	–0.066	0.072	−					
Degree	0.034	0.078	–0.075	−				
News media literacy	0.106	–0.003	0.044	–0.067	−			
Xenophobia	–0.033	–0.032	0.076	–0.037	−0.378**	−		
Blind patriotism	0.007	–0.048	0.036	–0.070	−0.362**	0.503**	−	
Intellectual humility	0.008	0.038	0.042	–0.111	0.345**	−0.344**	−0.271**	−
Perceived threat	0.057	–0.011	0.075	–0.067	−0.367**	0.507**	0.483**	−0.225**

**p < 0.001. The relationships between ordinal variables (degree and university) and other variables were calculated by Spearman’s rank correlation, while others were calculated by Pearson correlation.

### The measurement model

The measurement model of this study was examined through convergent validity and reliability (see Table 3). The standardized factor loadings of news media literacy, xenophobia, intellectual humility, blind patriotism, and perceived threat were greater than 0.5. The Cronbach’s alpha values were 0.94, 0.88, 0.89, 0.85. and 0.83, respectively. According to Taber (2018), a Cronbach’s alpha value above 0.7 is considered as indicating an acceptable level of reliability. The constructs also exhibited CR values greater than 0.7, and the AVE values exceeded the recommended threshold value of 0.5. Therefore, all the variables indicated satisfactory convergent validity and reliability.

**TABLE 3:** The item factor loadings, CR, Cronbach’s alpha values, and the instrument variable descriptive statistics (*N* = 430).

Instrument variable and measurement items	Factor loadings	Mean (*SD*)	α	AVE	CR
News media literacy (NML)	–	4.17 (1.474)	0.937	0.624	0.937
NML 1	0.802	4.37 (1.891)			
NML 2	0.752	4.27 (1.791)			
NML 3	0.814	4.07 (1.760)			
NML 4	0.736	4.16 (1.806)			
NML 5	0.766	4.34 (1.756)			
NML 6	0.823	4.08 (1.837)			
NML 7	0.794	4.21 (1.799)			
NML 8	0.808	4.11 (1.851)			
NML 9	0.808	3.93 (1.783)			
Xenophobia (XEN)	–	4.60 (1.422)	0.877	0.591	0.878
XEN 1	0.786	4.59 (1.813)			
XEN 2	0.695	4.48 (1.697)			
XEN 3	0.799	4.62 (1.704)			
XEN 4	0.743	4.72 (1.723)			
XEN 5	0.815	4.62 (1.744)			
Intellectual humility (IH)	–	4.16 (1.470)	0.884	0.559	0.884
IH 1	0.754	4.21 (1.846)			
IH 2	0.772	4.18 (1.827)			
IH 3	0.719	4.21 (1.841)			
IH 4	0.740	3.91 (1.888)			
IH 5	0.712	4.26 (1.854)			
IH 6	0.788	4.20 (1.837)			
Blind patriotism (BP)	–	4.42 (1.490)	0.847	0.583	0.848
BP 1	0.775	4.67 (1.799)			
BP 2	0.729	4.22 (1.815)			
BP 3	0.810	4.32 (1.826)			
BP 4	0.738	4.47 (1.739)			
Perceived threat (PT)	–	4.31 (1.461)	0.831	0.553	0.832
PT 1	0.758	4.49 (1.731)			
PT 2	0.741	4.13 (1.809)			
PT 3	0.710	4.33 (1.764)			
PT 4	0.764	4.30 (1.864)			

SD, Standard Deviation; AVE, Average Variance Extracted; CR, Composite Reliability.

In addition, regarding the goodness of fit for this model construct, χ^2^/*df* = 1.54, GFI = 0.92, AGFI = 0.91, IFI = 0.97, TLI = 0.97, CFI = 0.97, and RMSEA = 0.035 were acquired. The fit scale showed a sufficient fit for this model as these indices were approaching the criterion of a good fit according to some researchers (Bentler and Bonett, 1980; Bollen, 1989; West et al., 2012; Cangur and Ercan, 2015; Kline, 2015; Kim et al., 2019).

The discriminant validity was examined through HTMT. As shown in Table 4, all the values are below the stringent criterion of 0.80, as suggested by Henseler et al. (2015). This means that discriminant validity was also met. Therefore, according to the above results, all measures were found to be adequately valid and reliable, and both the convergent and construct validities were confirmed for all questionnaires in this model.

**TABLE 4:** Heterotrait-monotrait ratio of correlations result.

	Constructs	1	2	3	4	5
1	News media literacy	–				
2	Xenophobia	0.417	–			
3	Intellectual humility	0.406	0.583	–		
4	Blind patriotism	0.379	0.390	0.313	–	
5	Perceived threat	0.417	0.594	0.575	0.260	–

### The structural model

**FIGURE 2:**
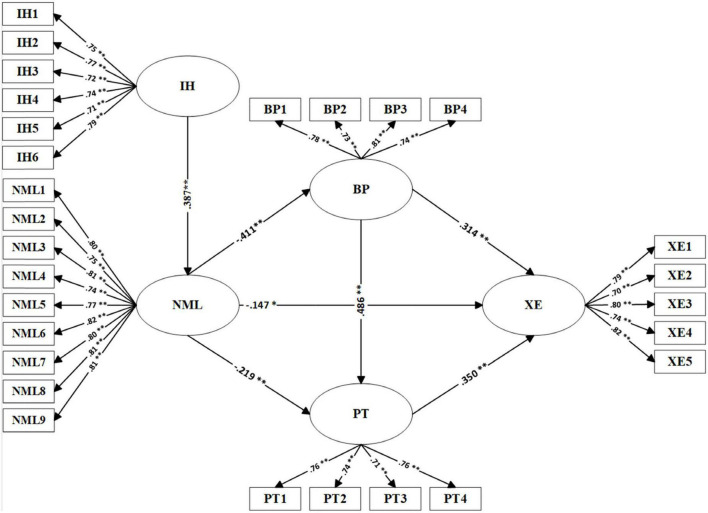
Structural path model.

The path analysis results are presented in Table 5 and Figure 2. Specifically, blind patriotism is positively associated with perceived threat (β = 0.486, *p* < 0.001), and both are positively correlated with xenophobia (β = 0.314 and 0.350, *p* < 0.001). As for news media literacy, it is negatively correlated with xenophobia (β = –0.147, *p* < 0.01), blind patriotism (β = –0.411, *p* < 0.001) and perceived threat (β = –0.219, *p* < 0.001). Intellectual humility was the positive predictor for explaining news media literacy (β = 0.387, *p* < 0.001). In summary, the above results indicate that the hypotheses in the model were all verified.

**TABLE 5:** Standardized regression weights according to the path model.

Path	*B*	β	*SE*	CR	*p*
News media literacy → Xenophobia	–0.138	–0.147	0.047	–2.947	< 0.01
News media literacy → Blind patriotism	–0.377	–0.411	0.050	–7.509	< 0.001
News media literacy → Perceived threat	–0.190	–0.219	0.047	–4.076	< 0.001
Blind patriotism → Perceived threat	0.458	0.486	0.058	7.840	< 0.001
Blind patriotism → Xenophobia	0.321	0.314	0.064	4.997	< 0.001
Perceived threat → Xenophobia	0.380	0.350	0.070	5.435	< 0.001
Intellectual humility → News media literacy	0.423	0.387	0.059	7.127	< 0.001

SE, Standard Error; CR, Composite Reliability.

In addition, χ^2^/*df*, AGFI, RMSEA, NNFI, CFI, and IFI were employed to assess the comprehensive goodness of fit of the research model (see Table 6). Results of structural equation modeling showed that the proposed model for the whole yielded a good fit.

**TABLE 6:** Model fit indices.

	Model	Acceptable values
χ^2^	549.813	
*P-*value	<0.001	
χ^2^/*df*	1.603	<3 (Kline, 2015)
AGFI	0.901	≥0.80 (Kim et al., 2019)
RMSEA	0.037	≤0.08 (Cangur and Ercan, 2015)
NNFI	0.966	≥0.90 (Bentler and Bonett, 1980)
CFI	0.969	≥0.95 (West et al., 2012)
IFI	0.970	≥0.90 (Bollen, 1989)

AGFI, Adjusted Goodness of Fit Index; RMSEA, Root Mean Square Error of Approximation; NNFI, Non-normed Fit Index; CFI, Comparative Fit Index; IFI, Incremental Fit Indices.

### Test of mediation

To further validate the structural relationships of our model, we assessed the serial mediation with blind patriotism and perceived threat serially mediating the relationship between news media literacy and xenophobia by plugin PROCESS 4.1 in SPSS 24.0 (see Table 7). The 95% confidence calculated by the tests showed that a mediating effect occurs when the range of intervals does not cross zero. The results showed that news media literacy had a significant indirect effect on xenophobia via blind patriotism [Effect = −0.10, *p* < 0.001, 95% CI = (−0.14, −0.07)], and news media literacy showed an indirect effect of −0.07 on xenophobia via perceived threat [*p* < 0.001, 95% CI = (−0.10, −0.04)]. Regarding the serial mediation analysis, we found a significant indirect effect serially via blind patriotism and perceived threat [Effect = −0.04, *p* < 0.001, 95% CI = (−0.06, −0.03)], and the path serially via blind patriotism and perceived threat contributed slightly more than the Ind 1 path but had no significant difference from the Ind 2 path. Thus, the mediation effects in the hypothesized model existed. In addition, as the direct effect of news media literacy on xenophobia is significant, the mediated effect and direct effect both exist and point in the same direction. According to Zhao et al. (2010), there is complementary partial serial mediation of blind patriotism and perceived threat on the relationship between news media literacy and xenophobia.

**TABLE 7:** Mediation result.

Parameter	Effect	BootSE	Lower	Upper	*p*
Ind1	–0.104	0.020	–0.144	–0.069	<0.001
Ind2	–0.065	0.017	–0.101	–0.035	<0.001
Ind3	–0.043	0.010	–0.063	–0.027	<0.001
Total	–0.212	0.028	–0.268	–0.160	<0.001

Ind1 = News media literacy → Blind patriotism → Xenophobia. Ind2 = News media literacy → Perceived threat → Xenophobia. Ind3 = News media literacy → Blind patriotism → Perceived threat → Xenophobia.

## Discussion and conclusion

News plays a role in establishing public agendas, while misinformation presented as news can contribute to severe social consequences and influence people’s perceptions of outgroup or specific events (Corser et al., 2022). Although there is increasing evidence for various media effects on public opinion, the relationship between news media and societal attitude is symbiotic. Societal attitudes such as xenophobia imply that such reports attract audiences. This would obviously affect the production of news as it affects the profits of news agencies (Hansen and Goligoski, 2018). Understanding this relationship is important, as reducing xenophobia may require effort regarding both sources. At the consumer end, cultivating news media literacy is important (Tully and Vraga, 2017). It helps individuals to access news content objectively to avoid media manipulation.

Although news media literacy has received much attention in the current complex media environments, and xenophobia has become more intensive with the Covid-19 pandemic, the relationship between the two factors among college students has rarely been explored, especially in the Asian context. As previous research has suggested, although contemporary young adults are always considered to be digital natives or cyber experts, they are vulnerable media users, which places them in a paradoxical situation (Livingstone and Helsper, 2010; De Leyn et al., 2022). Considering that students must be prepared for exposure to a media environment full of uncertainty, it is important to investigate the news media literacy and how it could combat xenophobia. In addition to this, it is also necessary to investigate factors that can serve as a predictor of news media literacy, which informs educators on how to cultivate news media literacy.

In this study, we investigated the relationship between news media literacy and xenophobia based on Chinese university students. Considering that news media literacy would affect how people obtain information, which further influences their perceptions of the outside world, this study also investigated the mediating role of blind patriotism and perceived threat. In addition, since news media literacy is about reviewing and processing information rationally, we also hypothesized that intellectual humility would be positively associated with news media literacy. Findings from 430 college students in China indicated that the relationship between news media literacy and xenophobia was mediated by blind patriotism and perceived threat. Specifically, people with higher news media literacy might perceive less threat and would not be so blindly patriotic. Therefore, they might have less xenophobic beliefs. In addition, blind patriotism was positively associated with perceived threat, and intellectual humility was a positive predictor of news media literacy. The results of this study are consistent not only with studies indicating that news media literacy affects individuals’ perceptions (Vraga et al., 2012), but also with studies which found that intellectual humility reliably predicts desire for authenticity (Koetke et al., 2022). Our findings suggested that to develop stronger news media literacy skills to combat xenophobia, educators may need to help cultivate students’ intellectual humility. In addition, blind patriotism and perceived threat are also two perspectives to understand xenophobia. The implications of this study are discussed below.

Firstly, this study employed Amos 24.0 to establish a valid and reliable five-variable structural equation model that measures the relationship between news media literacy and xenophobia among college students, and which integrates intellectual humility, blind patriotism, and perceived threat into the model. More interestingly, this study confirmed the mediating role of blind patriotism and perceived threat and the predicting role of intellectual humility. To the best of our knowledge, this is the first model that incorporates these variables to examine the relationship between news media literacy and xenophobia. Our model implies that the students’ news media literacy played a role in their acquisition and review of online information. Intellectual humility is significant (Whitcomb et al., 2017), and the traits it involves may be helpful for promoting news media literacy. Therefore, encouraging students to cultivate intellectual humility should be beneficial. However, students with perceived threat and blind patriotism may be discouraged from rationally perceiving the relationship with outgroups. Specifically, the results showed that people with low levels of news media literacy may perceive more threats and be more likely to be blindly patriotic, and therefore generate xenophobic beliefs. By comparing the effect size of each path, blind patriotism showed a stronger effect than perceived threat in the relationship between news media literacy and xenophobia.

The current results imply that the way students process information influences their views about outgroups, which also corresponds to the cognitive behavioral theory, indicating that one’s cognition would shape one’s behavior and vice versa (Potter, 2004). From a practical perspective, our survey findings from around 400 respondents can serve as useful input for courses on news media literacy. For example, educators should integrate communication strategies of news coverage and social media postings into the course to help students build a holistic view of the operation system of online information, to mitigate the negative psychological impacts of perceived threat and outgroup and ingroup relationships and to improve individual and societal wellbeing. Moreover, the results also imply the importance of some personal inner traits. For example, the inclusion of intellectual humility, rational perception of outgroup and ingroup relationships in the news media literacy education would help students to build a more scientific epistemology of news media literacy.

Secondly, this study enriches the research on news media literacy and xenophobia by broadening the samples to include data from China. Also, it helps better understand the news media literacy of college students from mainland China. Moreover, the results show that students with a higher level of news media literacy had positive perceptions of outgroup relationships, which is the same as the previous findings in western samples. It implies that news media literacy should also be promoted for Chinese students to reduce the chances of them developing negative outgroup perceptions.

Furthermore, Mason (2021) believed that Confucius started the work of outlining the concept of intellectual humility a long time ago. Therefore, it is necessary to explore it in the Chinese context. Within the Web of Science Core Collection database, we identified 382 studies containing the search term “intellectual humility.” However, further separate searches within this result using “Chinese” identified only one study (Wei and Wang, 2021). In this study, we noted that the mean score of students’ intellectual humility was also greater than the neutral point, and it was a significant predictor of news media literacy. This study could therefore further enrich the applicability of intellectual humility in the Chinese context and help gain a deeper understanding of this trait and virtue.

In conclusion, the emergence of the Internet and social media has changed the spread of information, and people must learn how to face this complex situation to avoid being emotionally affected by misinformation and disinformation. Students should develop news media literacy, which will form knowledge for their future decision making and allow them to examine information critically and to understand group relationships objectively. We also recommend that educators pay attention to the impact of intellectual humility on news media literacy and emphasize the danger of blind patriotism and perceived threats in the formation of xenophobia. This would help people to navigate an increasingly convoluted information ecosystem, promote harmonious relationships, and prevent violent extremism.

## Limitation

Study limitations and areas for continued research are important to note. Firstly, this study did not consider the influence of some underlying reasons, such as news bias and political bias. In addition, as Tully et al. (2020) suggested, understanding attitudes, motivations, and decision-making processes could also help to know more about news media literacy and its effects. More importantly, although some participants may have received news media literacy education before, we did not measure their achievements. Therefore, more empirical studies involving underlying factors and using longitudinal research designs are needed in future research. Thirdly, we acknowledge the limitations of the sample size and the lack of sample diversity. Future research can be expanded to other populations and larger groups in order to test the reasonability of our model. Furthermore, news media literacy includes far more than a set of skills that aim to identify misinformation—skills that will likely be made redundant or insufficient as technologies continue to develop, as the advent and application of new techniques is highly uncertain and unpredictable (Corser et al., 2022). In the increasingly complex information environment, the flaws of traditional media literacy have been shown (McGrew, 2020). Therefore, future research should continuously broaden the theoretical understanding and practical experience of news media literacy to keep people from falling into different traps.

## Data availability statement

The raw data supporting the conclusions of this article will be made available by the authors, without undue reservation.

## Ethics statement

Ethical review and approval was conducted by The Chinese University of Hong Kong (IRB Reference No. EDU2022-055). Written informed consent for participation was approved by The Chinese University of Hong Kong and signed by each participant in accordance with the national legislation and institutional requirements.

## Author contributions

ML and CC contributed to the conception and design of the study. ML performed the statistical analysis and wrote the first draft of the manuscript. CC and J-CL contributed to manuscript revision, read, and approved the submitted version. All authors contributed to the article and approved the submitted version.

## Footnotes

1
https://wj.qq.com

## References

[B1] Abdul M. Moch H. Aris F. Ahmad A. Ahmad H. (2019). “The effect of hate speech exposure on religious intolerance among indonesian Muslim teenagers,” in *Proceedings of the 2019 Ahmad Dahlan International Conference Series on Education & Learning, Social Science & Humanities (ADICS-ELSSH 2019)*, Amsterdam: Atlantis Press, 39-44. 10.2991/adics-elssh-19.2019.31

[B2] Alvarez-del Arco D. Monge S. Azcoaga A. Rio I. Hernando V. Gonzalez C. (2012). HIV testing and counselling for migrant populations living in high-income countries: A systematic review. *Eur. J. Public Health* 23 1039–1045. 10.1093/eurpub/cks130 23002238 PMC4051291

[B3] Arendt F. (2013). Dose-dependent media priming effects of stereotypic newspaper articles on implicit and explicit stereotypes. *J. Commun.* 63 830–851. 10.1111/jcom.12056

[B4] Ashley S. Maksl A. Craft S. (2013). Developing a news media literacy scale. *J. Mass Commun. Educ.* 68 7–21. 10.1177/1077695812469802

[B5] Barzilai S. Chinn C. A. (2020). A review of educational responses to the “post-truth” condition: Four lenses on “post-truth” problems. *Educ. Psychol.* 55 107–119. 10.1080/00461520.2020.1786388

[B6] Bentler P. M. Bonett D. G. (1980). Significance tests and goodness of fit in the analysis of covariance structures. *Psychol. Bull.* 88 588–606. 10.1037/0033-2909.88.3.588

[B7] Bergan D. Lee H. (2018). Media Literacy and response to terror news. *J. Media Lit. Educ.* 10 43–56. 10.23860/jmle-2018-10-03-03

[B8] Bollen K. A. (1989). A new incremental fit index for general structural equation models. *Soc. Methods Res.* 17 303–316. 10.1177/0049124189017003004

[B9] Born K. Edgington N. (2017). *Analysis of philanthropic opportunities to mitigate the disinformation/propaganda problem.* Menlo Park, CA: William and Flora Hewlett Foundation.

[B10] Bowes S. M. Costello T. H. Lee C. McElroy-Heltzel S. Davis D. E. Lilienfeld S. O. (2022). Stepping outside the echo chamber: Is intellectual humility associated with less political myside bias? *Pers. Soc. Psychol. Bull.* 48 150–164. 10.1177/0146167221997619 33719720

[B11] Bursztyn L. Egorov G. Enikolopov R. Petrova M. (2019). *Social media and xenophobia: Evidence from Russia*. Cambridge, MA: National Bureau of Economic Research. 10.3386/w26567

[B12] Calvillo D. P. Ross B. J. Garcia R. J. B. Smelter T. J. Rutchick A. M. (2020). Political ideology predicts perceptions of the threat of Covid-19 (and susceptibility to fake news about it). *Soc. Psychol. Pers. Sci.* 11 1119–1128. 10.1177/1948550620940539

[B13] Cangur S. Ercan I. (2015). Comparison of model fit indices used in structural equation modeling under multivariate normality. *J. Modern Appl. Statist. Methods* 14 152–167. 10.22237/jmasm/1430453580

[B14] Cheng Y. Lee C.-J. (2019). Online crisis communication in a post-truth Chinese society: Evidence from interdisciplinary literature. *Public Relat. Rev.* 45:101826. 10.1016/j.pubrev.2019.101826

[B15] Corser K. Dezuanni M. Notley T. (2022). How news media literacy is taught in Australian classrooms. *Austr. Educ. Res.* 49 761–777. 10.1007/s13384-021-00457-5 34276122 PMC8274262

[B16] De Leyn T. Waeterloos C. De Wolf R. Vanhaelewyn B. Ponnet K. De Marez L. (2022). Teenagers’ reflections on media literacy initiatives at school and everyday media literacy discourses. *J. Child. Media* 16 221–239. 10.1080/17482798.2021.1952463

[B17] De Master S. Le Roy M. K. (2000). Xenophobia and the European Union. *Comp. Polit.* 32 419–436. 10.2307/422387

[B18] de Zavala A. G. Cichocka A. Eidelson R. Jayawickreme N. (2009). Collective narcissism and its social consequences. *J. Pers. Soc. Psychol.* 97 1074–1096. 10.1037/a0016904 19968420

[B19] Deffler S. A. Leary M. R. Hoyle R. H. (2016). Knowing what you know: Intellectual humility and judgments of recognition memory. *Pers. Individ. Differ.* 96 255–259. 10.1016/j.paid.2016.03.016

[B20] Erba J. Chen Y. Kang H. (2019). Using media literacy to counter stereotypical images of Blacks and Latinos at a predominantly White university. *Howard J. Commun.* 30 1–22. 10.1080/10646175.2018.1423652

[B21] Erjavec K. Kovačič M. P. (2012). You don’t understand, this is a new war!” Analysis of hate speech in news web sites’ comments. *Mass Commun. Soc.* 15 899–920. 10.1080/15205436.2011.619679

[B22] Faulkner J. Schaller M. Park J. H. Duncan L. A. (2004). Evolved disease-avoidance mechanisms and contemporary xenophobic attitudes. *Group Proc. Intergroup Relat.* 7 333–353. 10.1177/1368430204046142

[B23] Finell E. Stevenson C. (2022). Interpersonal bonds with fellow nationals, blind patriotism and preference for immigrants’ acculturation. *Scand. J. Psychol.* 63 383–392. 10.1111/sjop.12817 35358329 PMC9544849

[B24] Fussell E. (2014). Warmth of the welcome: Attitudes toward immigrants and immigration policy. *Ann. Rev. Soc.* 40 479–498. 10.1146/annurev-soc-071913-043325 26966338 PMC4782982

[B25] Garipagaoglu C. Kilic H. Coskun Y. D. (2012). Pre-service teachers’ need for cognition. *Proc. Soc. Behav. Sci.* 55 148–154. 10.1016/j.sbspro.2012.09.488

[B26] Ghaddar A. Khandaqji S. Awad Z. Kansoun R. (2022). Conspiracy beliefs and vaccination intent for Covid-19 in an infodemic. *PLoS One* 17:e0261559. 10.1371/journal.pone.0261559 35020721 PMC8754330

[B27] Golan G. J. Waddell T. F. Barnidge M. (2021). Competing identity cues in the hostile media phenomenon: Source, nationalism, and perceived bias in news coverage of foreign affairs. *Mass Commun. Soc.* 24 676–700. 10.1080/15205436.2021.1884263

[B28] Gries P. H. Zhang Q. Crowson H. M. Cai H. (2011). Patriotism, nationalism and China’s US policy: Structures and consequences of Chinese national identity. *China Q.* 205 1–17. 10.1017/S0305741010001360

[B29] Guo L. (2020). China’s “fake news” problem: Exploring the spread of online rumors in the government-controlled news media. *Digital J.* 8 992–1010. 10.1080/21670811.2020.1766986

[B30] Hamada T. Shimizu M. Ebihara T. (2021). Good patriotism, social consideration, environmental problem cognition, and pro-environmental attitudes and behaviors: A cross-sectional study of Chinese attitudes. *SN Appl. Sci.* 3:361. 10.1007/s42452-021-04358-1

[B31] Han D. (2017). Is China ready for foreigners?: Public attitudes towards immigration in China. *China Int. J.* 15 120–143.

[B32] Hansen E. Goligoski E. (2018). *Guide to audience revenue and engagement. columbia journalism review.* Available online at: https://www.cjr.org/tow_center_reports/guide-to-audience-revenue-and-engagement.php (accessed October 2, 2022).

[B33] Henseler J. Ringle C. M. Sarstedt M. (2015). A new criterion for assessing discriminant validity in variance-based structural equation modeling. *J. Acad. Mark. Sci.* 43 115–135. 10.1007/s11747-014-0403-8

[B34] Hjerm M. (2001). Education, xenophobia and nationalism: A comparative analysis. *J. Ethnic Migr. Stud.* 27 37–60. 10.1080/13691830124482

[B35] Hobbs R. (2004). A review of school-based initiatives in media literacy education. *Am. Behav. Sci.* 48 42–59. 10.1177/0002764204267250

[B36] Huang W.-L. Liang J.-C. Tsai C.-C. (2017). Exploring the relationship between university. students’ conceptions of and approaches to learning mass communication in Taiwan. *Asia Pac. Educ. Res.* 27 43–54. 10.1007/s40299-017-0364-z

[B37] Ittefaq M. Abwao M. Baines A. Belmas G. Kamboh S. A. Figueroa E. J. (2022). A pandemic of hate: Social representations of Covid-19 in the media. *Anal. Soc. Issues Public Policy* 22 225–252. 10.1111/asap.12300

[B38] Jeong S.-H. Cho H. Hwang Y. (2012). Media literacy interventions: A meta-analytic review. *J. Commun.* 62 454–472. 10.1111/j.1460-2466.2012.01643.x 22736807 PMC3377317

[B39] Jones-Jang S. M. Mortensen T. Liu J. (2021). Does media literacy help identification of fake news? Information literacy helps, but other literacies don’t. *Am. Behav. Sci.* 65 371–388. 10.1177/0002764219869406

[B40] Kim Y. Lee S. Cho Y. Kim M. (2019). Analysis of causal relationships for nutrient removal of activated sludge process based on structural equation modeling approaches. *Appl. Sci.* 9:1398. 10.3390/app9071398

[B41] Kline R. B. (2015). *Principles and practice of structural equation modeling.* New York: Guilford publications.

[B42] Koetke J. Schumann K. Porter T. (2022). Intellectual humility predicts scrutiny of COVID-19 misinformation. *Soc. Psychol. Pers. Sci.* 13, 277–284. 10.1177/1948550620988242

[B43] Krumrei-Mancuso E. J. Rouse S. V. (2016). The development and validation of the comprehensive intellectual humility scale. *J. Pers. Assess.* 98 209–221. 10.1080/00223891.2015.1068174 26542408

[B44] Lajevardi N. Oskooii K. A. R. Walker H. (2022). Hate, amplified? Social media news consumption and support for anti-Muslim policies. *J. Public Policy* 1–28. 10.1017/S0143814X22000083 PMC11086701 38737869

[B45] Leary M. R. Diebels K. J. Davisson E. K. Jongman-Sereno K. P. Isherwood J. C. Raimi K. T. (2017). Cognitive and interpersonal features of intellectual humility. *Pers. Soc. Psychol. Bull.* 43 793–813. 10.1177/0146167217697695 28903672

[B46] Lee A. Y. L. Tiande W. (2016). “Teaching and learning media literacy in China: The uses of media literacy education,” in *Media Literacy Education in China*, ed. Cheung C.-K. (Singapore: Springer Singapore), 11–29.

[B47] Livingstone S. Helsper E. (2010). Balancing opportunities and risks in teenagers’ use of the internet: The role of online skills and internet self-efficacy. *New Media Soc.* 12 309–329. 10.1177/1461444809342697

[B48] Luo Y. F. Yang S. C. Kang S. (2022). New media literacy and news trustworthiness: An application of importance-performance analysis. *Comput. Educ.* 185:104529. 10.1016/j.compedu.2022.104529

[B49] Maksl A. Ashley S. Craft S. (2015). Measuring news media literacy. *J. Media Lit. Educ.* 6 29–45. 10.23860/jmle-6-3-3

[B50] Mamun M. A. Griffiths M. D. (2020). First Covid-19 suicide case in Bangladesh due to fear of Covid-19 and xenophobia: Possible suicide prevention strategies. *Asian J. Psychiatry* 51:102073. 10.1016/j.ajp.2020.102073 32278889 PMC7139250

[B51] Mason J. (2021). Confucius as an exemplar of intellectual humility. *J. Value Inq.* 10.1007/s10790-021-09806-0

[B52] McGrew S. (2020). Learning to evaluate: An intervention in civic online reasoning. *Comput. Educ.* 145:103711. 10.1016/j.compedu.2019.103711

[B53] Moore D. (2013). Bringing the world to school: Integrating news and media literacy in elementary classrooms. *J. Media Lit. Educ.* 5 326–336. 10.23860/jmle-5-1-5

[B54] Musgrove A. T. Powers J. R. Rebar L. C. Musgrove G. J. (2018). Real or fake? Resources for teaching college students how to identify fake news. *College Undergrad. Libr.* 25 243–260. 10.1080/10691316.2018.1480444

[B55] Newman D. Lewandowsky S. Mayo R. (2022). Believing in nothing and believing in everything: The underlying cognitive paradox of anti-Covid-19 vaccine attitudes. *Pers. Individ. Differ.* 189:111522. 10.1016/j.paid.2022.111522 35068637 PMC8761558

[B56] Noar S. M. (2003). The role of structural equation modeling in scale development. *Struct. Equa. Model. Multidiscip. J.* 10 622–647. 10.1207/S15328007SEM1004_8 33486653

[B57] Nshom E. Khalimzoda I. Sadaf S. Shaymardaov M. (2022). Perceived threat or perceived benefit? Immigrants’ perception of how Finns tend to perceive them. *Int. J. Intercult. Relat.* 86 46–55. 10.1016/j.ijintrel.2021.11.001

[B58] Onah E. I. (2008). The politics of xenophobia: Race, national groups and the anti-immigrant violence in South Africa. *IFE Psychol.* 16, 261–273. 10.4314/ifep.v16i3.23792

[B59] Parker C. S. (2010). Symbolic versus blind patriotism: Distinction without difference? *Polit. Res. Q.* 63 97–114. 10.1177/1065912908327228

[B60] Parker M. R. Foster L. N. Krohn K. R. Williams R. L. (2009). Relationship of College students’ patriotism to use of specific new sources and knowledge of current political events. *J. Polit. Mil. Soc.* 37 205–226.

[B61] Potter W. J. (2004). Argument for the need for a cognitive theory of media literacy. *Am. Behav. Sci.* 48 266–272. 10.1177/0002764204267274

[B62] Rydgren J. (2004). The logic of Xenophobia. *Rational. Soc.* 16 123–148. 10.1177/1043463104043712

[B63] Schatz R. T. (2020). “A review and integration of research on blind and constructive patriotism,” in *Handbook of patriotism*, ed. Sardoč M. (Cham: Springer). 10.1007/978-3-319-54484-7_30

[B64] Schatz R. T. Staub E. Lavine H. (1999). On the varieties of national attachment: Blind versus constructive patriotism. *Polit. Psychol.* 20 151–174. 10.1111/0162-895X.00140

[B65] Semyonov M. Raijman R. Tov A. Y. Schmidt P. (2004). Population size, perceived threat, and exclusion: A multiple-indicators analysis of attitudes toward foreigners in Germany. *Soc. Sci. Res.* 33 681–701. 10.1016/j.ssresearch.2003.11.003

[B66] Søe S. O. (2017). Algorithmic detection of misinformation and disinformation: Gricean perspectives. *J. Document.* 74 309–332. 10.1108/JD-05-2017-0075

[B67] Spry C. Hornsey M. (2007). The influence of blind and constructive patriotism on attitudes toward multiculturalism and immigration. *Austr. J. Psychol.* 59 151–158. 10.1080/00049530701449489

[B68] Stamps D. (2021). Media literacy as liberator: Black audiences’ adoption of media literacy, news media consumption, and perceptions of self and group members. *J. Int. Intercult. Commun.* 14 240–257. 10.1080/17513057.2020.1789692

[B69] Su Y. Lee D. K. L. Xiao X. (2022). I enjoy thinking critically, and I’m in control”: Examining the influences of media literacy factors on misperceptions amidst the Covid-19 infodemic. *Comput. Hum. Behav.* 128:107111. 10.1016/j.chb.2021.107111 34866771 PMC8631744

[B70] Suleman S. Garber K. D. Rutkow L. (2018). Xenophobia as a determinant of health: An integrative review. *J. Public Health Policy* 39 407–423. 10.1057/s41271-018-0140-1 30177729

[B71] Swart J. (2021). Tactics of news literacy: How young people access, evaluate, and engage with news on social media. *New Media Soc.* 1–17. 10.1177/14614448211011447

[B72] Taber K. S. (2018). The use of cronbach’s alpha when developing and reporting research instruments in science education. *Res. Sci. Educ.* 48 1273–1296. 10.1007/s11165-016-9602-2

[B73] Talwar S. Dhir A. Kaur P. Zafar N. Alrasheedy M. (2019). Why do people share fake news? Associations between the dark side of social media use and fake news sharing behavior. *J. Retail. Consum. Serv.* 51 72–82. 10.1016/j.jretconser.2019.05.026

[B74] Tamboer S. L. Kleemans M. Molenaar I. Bosse T. (2022). Developing a model of news literacy in early adolescents: A survey study. *Mass Commun. Soc.* 1–25. 10.1080/15205436.2022.2048027

[B75] The United and Nations Organization (2001). “Declaration on racism, discrimination, xenophobia and related intolerance against migrants and trafficked persons,” in *Presented at the Asia-Pacific NGO Meeting for the World Conference Against Racism, Racial Discrimination, Xenophobia and Related Intolerance*, (Teheran: The United Nations Organization).

[B76] Tucker J. A. Guess A. Barbera P. Vaccari C. Siegel A. Sanovich S. (2018). *Social media, political polarization, and political disinformation: A review of the scientific literature.* Menlo Park: William and Flora Hewlett Foundation.

[B77] Tully M. Vraga E. K. (2017). Effectiveness of a news media literacy advertisement in partisan versus nonpartisan online media contexts. *J. Broadcast. Electr. Media* 61 144–162. 10.1080/08838151.2016.1273923

[B78] Tully M. Vraga E. K. Smithson A.-B. (2020). News media literacy, perceptions of bias, and interpretation of news. *Journalism* 21 209–226. 10.1177/1464884918805262

[B79] van der Meer T. G. L. A. Hameleers M. (2022). I knew it, the world is falling apart! Combatting a confirmatory negativity bias in audiences’ news selection through news media literacy interventions. *Digital J.* 10 473–492. 10.1080/21670811.2021.2019074

[B80] van der Veer K. Yakushko O. Ommundsen R. Higler L. (2011). Cross-national measure of fear-based xenophobia: Development of a cumulative scale. *Psychol. Rep.* 109 27–42. 10.2466/07.17.Pr0.109.4.27-42 22049645

[B81] Vraga E. K. Tully M. Akin H. Rojas H. (2012). Modifying perceptions of hostility and credibility of news coverage of an environmental controversy through media literacy. *Journalism* 13 942–959. 10.1177/1464884912455906

[B82] Vromans L. Schweitzer R. D. Knoetze K. Kagee A. (2011). The experience of xenophobia in South Africa. *Am. J. Orthopsychiatry* 81 90-93. 21219279 10.1111/j.1939-0025.2010.01075.x

[B83] Wei X. Wang F. (2021). The influence of culture on wise reasoning in the context of self-friend conflict and its mechanism. *Acta Psychol. Sin.* 53:1244. 10.3724/SP.J.1041.2021.01244

[B84] West D. M. (2022). *How to combat fake news and disinformation. Brookings.* Available online at: https://www.brookings.edu/research/how-to-combat-fake-news-and-disinformation/ (accessed on October 3, 2022).

[B85] West S. G. Taylor A. B. Wu W. (2012). Model fit and model selection in structural equation modeling. *Handb. Struct. Equa. Model.* 1 209–231.

[B86] Whitcomb D. Battaly H. Baehr J. Howard-Snyder D. (2017). Intellectual humility: Owning our limitations. *Philos. Phenomenol. Res.* 94 509–539. 10.1111/phpr.12228

[B87] Willis-Esqueda C. Delgado R. H. Pedroza K. (2017). Patriotism and the impact on perceived threat and immigration attitudes. *J. Soc. Psychol.* 157 114–125. 10.1080/00224545.2016.1184125 27136269

[B88] Zhang D. (2021). Media and pollution in China: Mouthpiece or watchdog? *Int. J. Sustain. Policy Practice* 17:7. 10.18848/2325-1166/cgp

[B89] Zhao H. (2022). “Comparison of media literacy education between Chinese and Western Students,” in *Proceedings of the 2021 International Conference on Education, Language and Art (ICELA 2021)*, (Amsterdam: Atlantis Press), 295–298. 10.2991/assehr.k.220131.053

[B90] Zhao X. Lynch J. G. Jr. Chen Q. (2010). Reconsidering Baron and Kenny: Myths and truths about mediation analysis. *J. Consum. Res.* 37 197–206. 10.1086/651257

